# Training methods that improve MD–PhD student self-efficacy for clinical research skills

**DOI:** 10.1017/cts.2019.419

**Published:** 2019-10-14

**Authors:** Mathew Sebastian, Matthew A. Robinson, Leanne Dumeny, Kyle A. Dyson, Joseph C. Fantone, Wayne T. McCormack, W. Stratford May

**Affiliations:** 1 Lillian S. Wells Department of Neurosurgery, University of Florida College of Medicine, Gainesville, FL, USA; 2 MD-PhD Training Program, University of Florida College of Medicine, Gainesville, FL, USA; 3 Office of Research, University of Central Florida College of Medicine, Orlando, FL, USA; 4 Department of Pharmacotherapy and Translational Research, Genetics Institute, University of Florida College of Pharmacy, Gainesville, FL, USA; 5 Department of Pathology, Immunology & Laboratory Medicine, University of Florida College of Medicine, Gainesville, FL, USA; 6 Office of Biomedical Research Career Development, University of Florida Health Science Center, Gainesville, FL, USA; 7 Department of Medicine, Division of Hematology and Oncology, University of Florida College of Medicine, Gainesville, FL, USA

**Keywords:** Clinical research training, MD–PhD training, physician-scientist, biomedical research workforce, research self-efficacy

## Abstract

**Introduction:**

MD-PhD training programs train physician-scientists to pursue careers involving both clinical care and research, but decreasing numbers of physician-scientists stay engaged in clinical research. We sought to identify current clinical research training methods utilized by MD–PhD programs and to assess how effective they are in promoting self-efficacy for clinical research.

**Methods:**

The US MD–PhD students were surveyed in April–May 2018. Students identified the clinical research training methods they participated in, and self-efficacy in clinical research was determined using a modified 12-item Clinical Research Appraisal Inventory.

**Results:**

Responses were received from 61 of 108 MD–PhD institutions. Responses were obtained from 647 MD–PhD students in all years of training. The primary methods of clinical research training included no clinical research training, and various combinations of didactics, mentored clinical research, and a clinical research practicum. Students with didactics plus mentored clinical research had similar self-efficacy as those with didactics plus clinical research practicum. Training activities that differentiated students who did and did not have the clinical research practicum experience and were associated with higher self-efficacy included exposure to Institutional Review Boards and participation in human subject recruitment.

**Conclusions:**

A clinical research practicum was found to be an effective option for MD–PhD students conducting basic science research to gain experience in clinical research skills. Clinical research self-efficacy was correlated with the amount of clinical research training and specific clinical research tasks, which may inform curriculum development for a variety of clinical and translational research training programs, for example, MD–PhD, TL1, and KL2.

## Introduction

The core mission of MD–PhD training programs is to train physician-scientists to be translators of benchtop discoveries into clinical research advances [1]. Concern has been expressed over the past several decades about the overall attrition in the physician-scientist workforce, and the research career challenges faced by physician-scientists have been described [2,3]. Common challenges include administrative hurdles, obtaining Institutional Review Board (IRB) approval, difficulty of subject recruitment, administering complicated informed consent agreements, securing protected time for investigators, and completing large amounts of regulatory paperwork [4,5]. Without building self-efficacy (confidence) for skills necessary to conduct clinical research, trainees and early career stage physician-scientists may not choose to pursue career pathways that utilize their combined clinical and research training skills [6]. Little is known about the methods used by MD–PhD programs to provide training in clinical research and how to overcome barriers to conducting clinical research.

One strategy for enhancing the engagement of MD–PhD trainees in clinical research is to provide clinical research experiences during training that develop self-efficacy for these research skills. Self-efficacy theory posits that those who are confident in their ability to complete a specific task are more likely to engage in that behavior and are more likely to persist despite obstacles that may arise [7,8]. Social cognitive career theory highlights self-efficacy as a major factor influencing career choices and decisions, suggesting that trainees possessing high confidence in their clinical research skills are more likely to pursue careers in clinical research [9]. Although self-efficacy is generally accepted to be a positive predictor of scholarly productivity and research interest [10,11], studies have shown that the positive relationship between self-efficacy and performance may partially be explained as a function of performance’s influence on self-efficacy and not the other way around [12]. Additionally, other studies have concluded that self-efficacy leads to overconfidence which, in return, leads to a negative relationship between performance and self-efficacy [13,14]. Although confidence in performing a given task may not equate directly with the actual ability to perform the task, increased confidence in clinical research is evidence that MD–PhD programs are at least exposing students to clinical research. Assessment tools that measure clinical research self-efficacy may then be useful to evaluate the impact of research training programs’ curricula in promoting the development of future physician-scientists who focus on clinical research.

MD–PhD curricula are constantly evolving to integrate clinical and research training within educational environments that traditionally separate medical and graduate research training. Curricula designed to promote cognitive integration, connecting distinct domains of knowledge to achieve conceptual coherence, have been demonstrated to improve transfer, reasoning, and future learning [15–18]. In order to achieve such integration of clinical and research training, some programs have adopted clinical research practicum courses, that is, experiential learning programs, in which students design and carry out a hypothesis-driven, supervised investigation involving human subjects. In this study, we assess the methods currently used by MD–PhD programs for clinical research training alone or in combinations, that is, didactic courses, mentored clinical research, and clinical practicum courses. Furthermore, we investigate the effect of these training methods on student self-efficacy relating to several measures important for clinical research. Through distribution by MD–PhD directors and coordinators, we invited MD–PhD students nationwide to participate in an anonymous, web-based survey to determine what their clinical research training experiences have been and utilized the Clinical Research Appraisal Inventory (CRAI) to assess student’s self-efficacy in items important for clinical research [19]. We hypothesized that students who received hands-on training in clinical research, such as mentored clinical research for dissertation work or a clinical research practicum, would have greater self-efficacy in several characteristics of clinical research as compared with those who had not received clinical research training or received didactic training alone.

## Materials and Methods

### Study Design

Of the 127 US MD–PhD Training Institutions listed by the Association of American Medical Colleges, 108 were found to have functional MD–PhD Program webpages and/or institutional email addresses [20]. Program directors and coordinators were emailed an anonymous online survey link via Qualtrics Survey Software (Qualtrics, Provo, UT) and asked to forward the survey information to their students (Supplementary material Appendix S1). Directors were sent two reminder emails. No compensation was provided for this study. Data were collected from April 1st to May 31st, 2018. Participants consented on the first page of the Qualtrics Survey prior to starting the survey. The survey asked for the name of their current institution, year of training, and method of clinical research training they had received as an MD–PhD student, with the following options: (1) none, (2) didactic coursework, (3) formal student practicum program, (4) clinical research training by a primary research mentor, and/or (5) other (specified by the student). Clinical research was defined as research involving human participants either through direct interaction or through the collection and analysis of blood, tissues, or other samples. A clinical research practicum was defined as experiential learning that included a hypothesis-driven, supervised investigation involving human subjects that implements a clinical research design. This study was approved as exempt by the University of Florida IRB (IRB201800047) on February 15, 2018. This study did not receive outside funding.

### Measures

The CRAI was designed to assess the self-efficacy of students in performing different aspects of clinical research. A modified version of the 12-item CRAI was used to evaluate students in six domains aligned with skills for conducting clinical research: Planning; Designing and Collecting; Funding; Conceptualizing and Collaborating; Protecting; Reporting, Interpreting, and Presenting [19]. The inventory in this study was rated on a 1–10 scale (1 = no confidence to 10 = total confidence).

### Statistical Analysis

We categorized the free-text answers and analyzed the responses using descriptive statistics. All tests were two-sided, and significance was defined as *P* < 0.05. Tukey’s multiple comparisons of means tests were used to determine significant differences between clinical research training methods. Z-score tests for two population proportions were used to determine differences in tasks completed among different clinical research training methods. Unpaired *t* tests using the Bonferroni–Dunn method for multiple comparisons were used to assess differences in self-efficacy in the clinical research domains among students completing research tasks that were likely to involve direct human subject interaction (IRB submission, IRB approval, subject recruitment) versus research tasks that do not necessarily involve direct human subject interaction (data collection, data analysis, publication). Cronbach’s alpha tests were used to assess the internal consistency of the instrument. Descriptive statistics and figures were generated using Prism 7 (Version 7, GraphPad Software, La Jolla, CA).

## Results

### Participation

Responses were obtained from 61 of the 108 medical schools contacted, leading to an institutional response rate of 56.5%. The geographic census regions of the participating MD–PhD institutions in this study closely represent the total MD–PhD institution distribution among US geographical census regions (Table 1). The participating programs collectively enroll approximately 65.7% of the estimated 5494 MD–PhD students currently in training [21]. Of the 61 institutions that responded, 37 had Clinical and Translational Science Awards (CTSA) and 32 had Medical Scientist Training Program awards. Of the 647 participants who provided informed consent, 613 identified their year in program, 594 identified the type of clinical research training they have received, 504 participants answered at least one CRAI question, and 500 answered all 12 CRAI questions. With a total of 3613 MD–PhD students at the 61 medical schools represented [21], the response rate was 17.9%, with 13.8% completing the entire survey.

**Table 1.** Demographics of MD–PhD respondents to a survey regarding clinical research training methods and self-efficacy in clinical research in the USA, 2018

Program characteristics	Number of MD–PhD programs (%)	
Census region	In this study	Total^a^
West	10 (16.3)	18 (14.1)
Midwest	13 (21.3)	34 (26.8)
South	21 (34.4)	45 (35.4)
Northeast	17 (27.8)	30 (23.6)
Total	61	127
**Student characteristics**	**Number of participants (%)**	
	**In this study**	**Total**
Total participants (agreed to informed consent)	647 (17.9)	3,613^b^
Identified year of training	613 (94.7)	
Identified clinical research training method	594 (91.8)	
Answered all 12 CRAI questions	500 (77.3)	
**Year of study**	**In this study**	**95% CI of proportion**
MS1	92 (15.0)	12.3–18.1
MS2	51 (8.3)	6.3–10.8
GS1	103 (16.8)	13.9–20.0
GS2	67 (10.9)	8.6–13.7
GS3	77 (12.6)	10.0–15.4
GS4	77 (12.6)	10.0–15.4
GS5–GS7	19 (3.0)	1.9–4.8
MS3	59 (9.6)	7.4–12.2
MS4	68 (11)	8.7–13.9
Total	613	

CRAI, Clinical Research Appraisal Inventory; CI, confidence interval; MS1, Medical School Year 1; MS2, Medical School Year 2; GS1, Graduate School Year 1; GS2, Graduate School Year 2; GS3, Graduate School Year 3; GS4, Graduate School Year 4; GS5, Graduate School Year 5; GS6, Graduate School Year 6; GS7, Graduate School Year 7; MS3, Medical School Year 3; MS4, Medical School Year 4.

a Derived from Association of American Medical Colleges data (https://students-residents.aamc.org/applying-medical-school/article/mdphd-degree-programs-state/; accessed March 5, 2018).

b Derived from Association of American Medical Colleges data (https://www.aamc.org/download/321554/data/factstableb11-2.pdf; accessed September 17, 2018).

### Clinical Research Training Methods Used

First, we assessed the various training methods currently used by MD–PhD programs to train students in clinical research (Table 2). Of the 594 students who responded to this question, 150 (25.3%) responded saying they have received no training in clinical research, 194 (32.7%) respondents had completed didactic coursework, and 70 (11.8%) respondents said they were doing clinical research with their primary research mentor. All other responses were combinations of clinical research training methods: 106 (17.8%) of respondents experiencing didactics and mentored clinical research; 22 (3.7%) respondents experiencing didactics plus clinical research practicum; 4 (0.7%) have had mentored clinical research plus a clinical research practicum; and 48 (8.1%) of respondents experienced didactics, mentored clinical research, and a clinical research practicum. Because year in program influences the opportunity for clinical research training, participation in each training method was determined by year (Supplementary material Appendix S2). By the end of their first year of training (Medical School Year 1 (MS1)), 39.5% (34 out of 86) of students had received no clinical research training and 37.2% (32 out of 86) had received only didactic coursework with the remaining 23.3% (20 out of 86) students having participated in hands-on clinical research training, that is, clinical research practicum and/or mentored clinical research. At the end of the last year of training (Medical School Year 4 (MS4)), 16.4% (11 out of 67) of students had received no clinical research training and 26.9% (18 out of 67) had received didactic coursework, with 56.7% (38 out of 67) participating in hands-on clinical research experiences. For the following analyses, the training method with the smallest number of respondents (*n* = 4) was excluded from the analysis due to the low sample size.

**Table 2.** Clinical research training methods experienced by MD–PhD students in the USA, 2018

Clinical research training method	No. In this study (%)	95% CI of proportion
None (N)	150 (25.3)	21.8–28.9
Didactics (D) only	194 (32.7)	28.9–36.6
Mentored Clinical Research (R) Only	70 (11.8)	9.3–14.7
RP	4 (0.7)	0.2–1.7
DR	106 (17.8)	14.8–21.2
DP	22 (3.7)	2.3–5.6
DRP	48 (8.1)	6.0–10.6
Total	594 (100)	

CI, confidence interval; RP, mentored clinical research and clinical research practicum; DR, didactics and mentored clinical research; DP, didactics and clinical research practicum; DRP, didactics, mentored clinical research, and clinical research practicum. The clinical research training methods MD–PhD students in all years of training have experienced, 2018.

### Effect of Clinical Research Training Methods on Self-Efficacy for Clinical Research

**Fig. 1.**
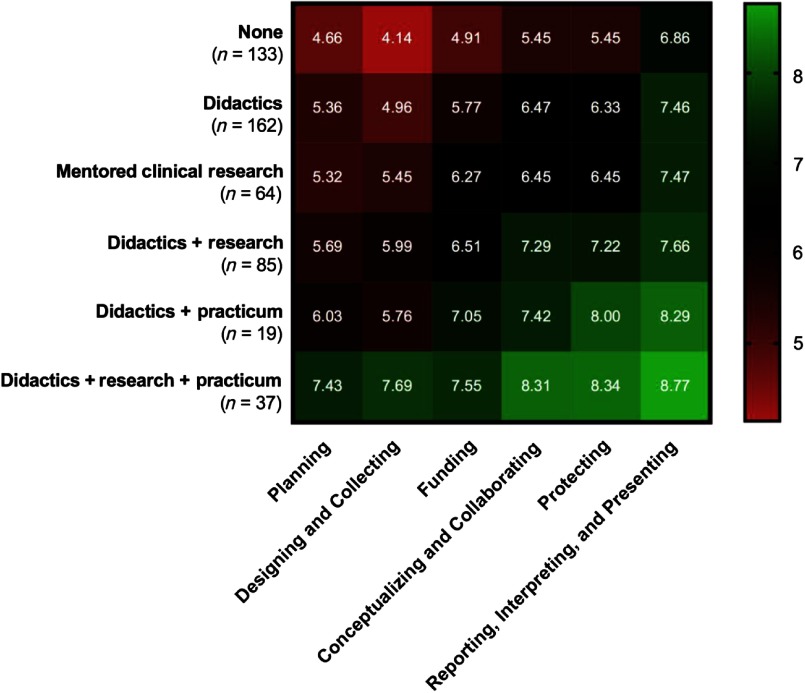
Self-efficacy scores for clinical research training methods experienced by MD–PhD students.

*Note:* The figure shows differences in self-efficacy scores among different clinical research training methods among 500 MD–PhD students in the USA, 2018. The score range is 1–10; 1, no confidence; 10, total confidence.

Self-efficacy for clinical research was assessed using the CRAI (scale 1–10; 1, no confidence; 10, total confidence, Supplementary material Appendix S1). Self-efficacy scores for six domains of clinical research skills are shown in Table 3. The mean values for different clinical research training methods are compared in Fig. 1. Students engaged in didactics, mentored clinical research, plus a clinical research practicum had the highest overall average scores among all six domains. Similar scores were observed between students engaged in didactics plus mentored clinical research versus didactics plus a clinical research practicum. Similarly, a large overlap was seen in students who only engaged in mentored clinical research and those who received didactics alone. All groups who received any clinical research training had higher self-efficacy scores compared to students with no training. Tukey’s multiple comparisons of means tests were used to identify differences between clinical research training methods (Table 4). Mean scores were significantly higher for most single or combination clinical research training methods compared to no training. No significant differences were observed for didactics alone versus mentored clinical research alone. No significant differences were observed for didactics plus clinical research practicum versus didactics plus mentored clinical research.

**Table 3.** Self-efficacy scores for clinical research training methods experienced by MD–PhD students in the USA, 2018

	Clinical research domains					
Training method	Planning	Designing and collecting	Funding	Conceptualizing and collaborating	Protecting	Reporting, interpreting, and presenting
None (*n* = 133)						
Mean (SD)	4.66 (2.3)	4.14 (2.0)	4.91 (2.5)	5.45 (1.9)	5.45 (2.1)	6.86 (2.2)
95% CI	4.27–5.06	3.79–4.49	4.49–5.33	5.12–5.78	5.08–5.81	6.48–7.24
Didactics only (*n* = 162)						
Mean (SD)	5.36 (2.2)	4.96 (1.9)	5.77 (2.3)	6.47 (1.5)	6.33 (2.0)	7.46 (1.7)
95% CI	5.01–5.70	4.67–5.24	5.42–6.12	6.24–6.71	6.02–6.64	7.19–7.73
Mentored clinical research (*n* = 64)						
Mean (SD)	5.32 (1.8)	5.45 (2.4)	6.27 (1.8)	6.45 (1.9)	6.45 (2.2)	7.47 (1.8)
95% CI	4.88–5.76	4.86–6.03	5.81–6.72	5.99–6.92	5.90–7.01	7.01–7.92
Didactics + mentored clinical research (*n* = 85)						
Mean (SD)	5.69 (1.9)	5.99 (2.0)	6.51 (2.2)	7.29 (1.6)	7.22 (1.8)	7.66 (1.7)
95% CI	5.29–6.01	5.56–6.42	6.05–6.98	6.94–7.64	6.84–7.61	7.30–8.01
Didactics + practicum (*n* = 19)						
Mean (SD)	6.03 (1.9)	5.76 (1.9)	7.05 (2.3)	7.42 (1.8)	8.00 (1.4)	8.29 (1.7)
95% CI	5.10–6.95	4.86–6.67	5.95–8.15	6.58–8.27	7.34–8.66	7.48–9.10
Didactics + mentored clinical research + practicum (*n* = 37)						
Mean (SD)	7.43 (1.6)	7.69 (1.7)	7.55 (1.9)	8.31 (1.3)	8.34 (1.6)	8.77 (1.0)
95% CI	6.91–7.95	7.13–8.25	6.92–8.19	7.88–8.75	7.82–8.85	8.45–9.09

SD, standard deviation; CI, confidence interval.

Self-efficacy scores of participating MD–PhD students in all years of training. The score range is 1–10; higher scores indicate greater self-efficacy.

**Table 4.** Comparisons of reported self-efficacy among clinical research training methods

Clinical research training methods		Clinical research domains					
Method 1	Method 2	Planning	Designing and collecting	Funding	Conceptualizing and collaborating	Protecting	Reporting, interpreting, and presenting
DRP	N	**	**	**	**	**	**
	D	**	**	**	**	**	**
	R	**	**	ns	**	**	*
	DR	**	**	ns	*	*	*
	DP	ns	*	ns	ns	ns	ns
DP	N	ns	*	**	**	**	*
	D	ns	ns	ns	ns	*	ns
	R	ns	ns	ns	ns	*	ns
	DR	ns	ns	ns	ns	ns	ns
DR	N	*	**	**	**	**	*
	D	ns	*	ns	*	*	ns
	R	ns	ns	ns	*	ns	ns
R	N	ns	**	**	*	*	ns
	D	ns	ns	ns	ns	ns	ns
D	N	ns	*	*	**	*	ns

N, none; D, didactics; R, mentored clinical research; DR, didactics plus mentored clinical research; DP, didactics plus clinical research practicum; DRP, didactics plus mentored clinical research plus clinical research practicum; ns, not significant, *P < 0.05, **P < 0.001.

### Clinical Research Self-Efficacy Increases During MD–PhD Training

**Fig. 2.**
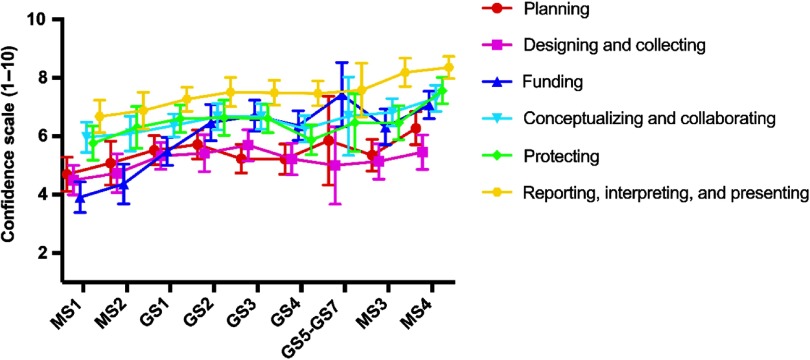
Self-efficacy progression for clinical research through MD–PhD training.

*Note:* Abbreviations as in Table 1. Data derived MD–PhD students in all years of training. The score range is 1–10; higher scores indicate greater self-efficacy. Data points represent means. Error bars indicate 95% confidence intervals.

Next, self-efficacy was assessed as students progress through the MD–PhD program. When comparing the first-year medical students (MS1 – first year of program) with the fourth-year medical students (MS4 – last year of program), self-efficacy for clinical research skills increased by an average of 1.75 in all domains of the CRAI, with the smallest increase being in the Designing and Collecting (0.96) and the largest being in Funding (3.16) (Fig. 2). All measures peaked in the last year of medical school except for two, (1) Funding and (2) Designing and Collecting, which both peaked during the graduate school phase of the program.

### Clinical Research Tasks Positively Associated with Clinical Research Self-Efficacy

**Fig. 3.**
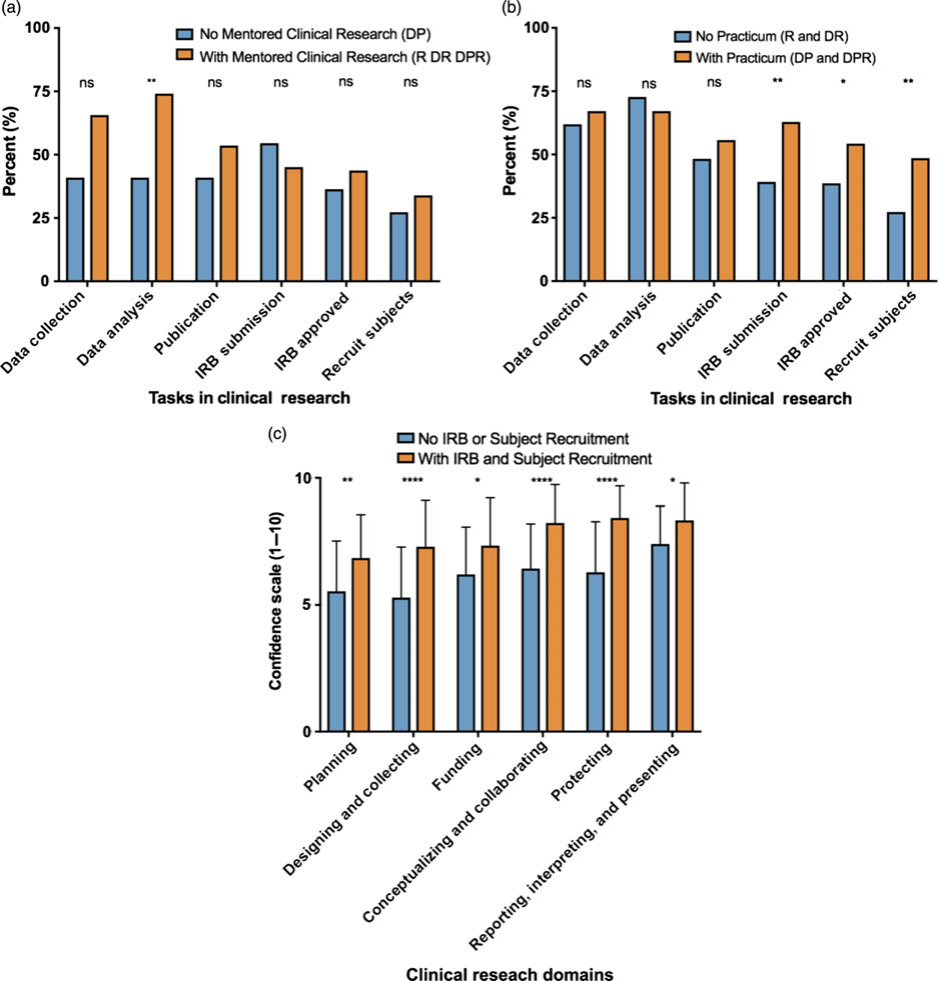
Clinical research tasks completed during mentored clinical research and/or clinical research practicum experiences.

*Note:* Abbreviations: R, mentored clinical research; DR, didactics plus mentored clinical research; DP, didactics plus clinical research practicum; DPR, didactics plus clinical research practicum plus mentored clinical research. The figure shows differences in clinical research tasks completed by 190 MD–PhD Students in the USA in 2018 in (a) No mentored clinical research (DP) versus with mentored clinical research (R, DR, DPR) or (b) No practicum (R, DR) versus with Practicum (DP, DPR). (c) Clinical research self-efficacy for 48 MD–PhD students who reported no experience with IRB submission, IRB approval, or subject recruitment, three research tasks most closely associated with direct human subject interaction (No IRB or Subject Recruitment) versus 48 MD–PhD students who reported experience with all three research tasks associated with direct human subject interaction and training (With IRB and Subject Recruitment). *P < 0.05, **P < 0.005, ****P < 0.0001.

In an effort to assess how mentored clinical research and a clinical research practicum may have impacted self-efficacy, students who experienced either or both of these training methods were asked to identify which clinical research tasks they had completed during training (data collection, data analysis, publication, IRB submission, IRB approval, or subject recruitment). When students with and without mentored clinical research were compared, data analysis was the only clinical research skill for which a significantly higher percentage of students had experienced due to mentored clinical research (*P* = 0.001; 166 out of 224 [74.1%] vs. 9 out of 22 [40.9%]), Fig. 3a). When students with and without a clinical research practicum were compared, a significantly higher proportion of students with the practicum had completed an IRB submission (*P* < 0.01; 44 out of 70 [62.9%] vs. 69 out of 176 [39.2%]), had an IRB approval (*P* = 0.03; 38 out of 70 [54.3%] vs. 68 out of 176 [38.6%]), and helped recruit participants to studies (*P* < 0.01; 34 out of 70 [48.6%] vs. 48 out of 176 [27.3%]) (Fig. 3b).

These results reveal some differences between the research tasks that are commonly performed during mentored clinical research and clinical research practica. Tasks that are more likely to involve human subjects interaction and training (IRB submission, IRB approval, and subject recruitment) appear to be more common with clinical research practica. The next question is whether these research tasks may have an impact on clinical research self-efficacy. As shown in Fig. 3c, experience with all three research tasks that are most associated with direct human subject interaction and training (IRB submission, IRB approved, subject recruitment) is associated with significantly higher clinical research self-efficacy scores than experience with any combination of research tasks that do not necessarily involve human subject interaction and training (data collection, data analysis, publication).

## Discussion

A critical question about the training of physician-scientists is whether it is important to expose MD–PhD students to clinical research during or after their MD–PhD training. During the survey period, some MD–PhD directors questioned the utility of these approaches, whereas others asked for additional information or shared their clinical research training curricula. However, the MD–PhD Program Outcomes Study concluded that “… the current number of MD–PhD program graduates per year will not meet expected workforce needs… As a result, other approaches to training physician-scientists have been and will continue to be required…” [22]. Because various clinical research training approaches for MD–PhD students exist, we believe there is equipoise on the topic to warrant further investigation as to whether higher self-efficacy at this early stage of training predicts participation in clinical research after completion of postgraduate training.

MD–PhD students are expected to be the future translators of basic science discoveries to advances in patient care, yet, at the end of their training, 43.3% (29 out of 67) of surveyed students reported having no hands-on clinical research training (Supplementary material Appendix S2). This included 26.9% (18 out of 67) with didactic training only and 16.4% (11 out of 67) with no clinical research training at all. Although some clinical research training occurs during postgraduate residency and fellowship training, this is also a period with limited protected research time and demanding clinical obligations with relatively fixed training requirements to achieve competency in foundational clinical disciplines irrespective of the career trajectory of the trainee [17,18]. Early clinical research interventions during the more flexible MD–PhD training build foundational knowledge in a protected and supervised environment for students to learn and obtain experience, even before residency training, connecting the two realms of their future: advancing clinical knowledge through relevant research inquiry.

Ng *et al.* argued that successful integrated training for physician-scientists requires development of at least three traits: cognitive synergy, sense of self, and professional capacity [23]. Clinical research practica and mentored clinical research synergize and function to integrate the clinical and research realms, enabling students to ask clinically relevant questions to be answered by sound research planning. These hands-on clinical research experiences may also facilitate development of a unique sense of self as a physician-scientist by placing students in a role as leaders of an interdisciplinary team. Participating in hands-on clinical research early in training may serve to motivate students and instill many of the aptitudes necessary of physician-scientists to develop into professional clinical researchers. Furthermore, these experiences are aligned with the program objective of the National Institutes of Health T32 Medical Scientist Training Program to encourage “…changes in integrated medical and graduate training that keep pace with the rapid evolution of a research environment that is increasingly complex, interdisciplinary, quantitative, and collaborative” [1].

Formal MD–PhD curricula require careful planning and institutional commitment. Clinical practicum requirements and length vary by program. Although some programs that offer a practicum require students to observe mentors going through the motions of clinical research, others actively require students to write an IRB protocol and attend IRB meetings, participate in clinical research team meetings, work with a research coordinator, collaborate with a biostatistician, participate in the recruitment and consent of study subjects, and shadow physicians enrolling study participants [24]. Additionally, the length of a clinical research practicum varies by program. Based on a review of MD–PhD websites, some programs use clinical research practica as a longitudinal study throughout multiple years of MD–PhD training, starting the first year of medical school and often continuing through the graduate school years. Others offer clinical research practica as mandatory or elective training during the traditionally clinical fourth year of medical study, which may be open to all medical students as a way to increase physician-scientist numbers.

Many combined degree programs have proposed alternatives to improve the success rate of training physician-scientists with a clinical/translational science focus [25–27]. Our findings reveal that both mentored clinical research and clinical research practica improve self-efficacy for clinical research skills, which in turn may support retention of physician-scientists in research careers involving clinical studies. A formalized clinical research practicum may also be especially helpful for MD–PhD students conducting basic science research. Such students may gain clinical research experience and foster translation of their research to the clinical setting because those participating in a clinical research practicum have greater experience with the planning and recruitment stages of the clinical research process (Fig. 3b). In addition, these training methods may also apply to MD-only graduates during postdoctoral residency or fellowship training in order to increase physician-scientist numbers.

One of the challenges faced in training a future physician-scientist workforce is the relatively high attrition rate within MD–PhD programs. As pointed out by Jeffe *et al.*, approximately 27% of incoming MD–PhD students either graduate with an MD degree only or no degree [28]. This, compounded with the rigors of MD–PhD programs, may lead some trainees to forgo careers involving clinical research. Here, we have shown that exposure of trainees to clinical research training experiences is correlated with significantly greater self-efficacy for clinical research skills that will hopefully translate to increased engagement and retention of physician-scientists in clinical research. Such training may decrease the attrition rate among MD–PhD students by offering enhanced clinical relevance to the research career of a physician-scientist. These findings may also inform the design of training programs for other learners in the pipeline for the clinical and translational research workforce, such as PhD students, professional and dual degree students, and postdoctoral fellows in CTSA TL1 training programs, and early-stage investigators in CTSA KL2 training programs.

### Limitations of this Study

This is the first study to assess the state of clinical research training among all US MD–PhD training programs. Limitations of this study include the use of self-efficacy as a predictor of later involvement clinical research. Although a plethora of studies support self-efficacy theory, and the way it is used in this study, other studies question the theory, suggesting that more work needs to be done in the field. Although some studies show that confidence may not directly equate to performance and interest, in this context, it can be used to indicate the exposure a student has had to clinical research during the tenure of their MD–PhD training.

A major limitation of this study is the low response rate. Although 17.9% of students agreed to the informed consent, 13.8% completed the whole survey. Few programs have experiential learning programs, limiting the possible number of respondents with this training. Additionally, these data are from only 1 year of study, and data received from MD–PhD students in other institutions and years may differ. Further research should be directed at follow-up of graduates’ career focus based on clinical research educational experiences and examine subsequent years of MD–PhD students to assess whether there are changes in choice of career paths over time in cohorts of MD–PhD students. In this study, name and contact information were not collected in order to preserve anonymity, so it will not be possible to correlate findings with career outcomes. Because these data are self-reported, they may be subject to social desirability bias.

The definition of clinical research used in this study can be interpreted to include MD–PhD students with no research subject interaction and training (e.g., analysis of human samples only), with research subject interaction (recruitment, enrollment, and consent), or both. Interestingly, research tasks during training that do not necessarily involve direct human interaction and training (data collection, data analysis, publication) do not appear to improve clinical research self-efficacy as well as tasks that most likely involve direct human interaction and training (IRB submission, IRB approval, subject recruitment) (Fig. 3c), suggesting that human subjects-oriented research tasks and training may be a critical elements for effective clinical research training.

## Conclusions

Health care needs physician-scientists who can apply both foundational biomedical knowledge and clinical research skills to advance the practice of medicine. MD–PhD students with more diverse clinical research training experiences had increased self-efficacy in various clinical research domains. MD–PhD students who are not pursuing clinical research for their doctoral research may attain a similarly high level of clinical research self-efficacy when they participate in a clinical research practicum experience.

## Supplementary material

For supplementary material accompanying this paper visit https://doi.org/10.1017/cts.2019.419. click here to view supplementary material
