# Supplementary material for: Training methods that improve MD–PhD student self-efficacy for clinical research skills
Source: J Clin Transl Sci. 2019 Oct 14;3(6):316–24. doi: 10.1017/cts.2019.419 (PMC6885998; doi:10.1017/cts.2019.419)
Supplement: Supplementary file 1 [file S2059866119004199sup001.docx]

**Supporting Information**

**Appendix S1.** Survey Design.

**Appendix S2.** Proportion of clinical research training formats experienced by students in all years of training.

**Appendix S1.** Survey Design.

| **Survey Questions** | | |
| --- | --- | --- |
| Where are you currently conducting your MD-PhD training? (Please enter whole name) | | |
| What year are you?   Response options: MS1; MS2; GS1; GS2; GS3; GS4; GS5; MS3; MS4; Other | | |
| What kind of formal clinical research training have you received as an MD-PhD student? Check all that apply. Definitions: 1) clinical research- research involving human participants either through direct interaction or through the collection and analysis of blood, tissues, or other samples 2) Practicum- an experiential learning, hypothesis-driven, supervised investigation involving human subjects that applies and implements a clinical research design. | | |
| Response options: None; Didactic coursework; Formal student practicum program; Research training through primary mentor; Other | | |
| (If 'Formal student practicum program' was not marked) Would you be interested in having a formal practicum program where as students, you develop and complete a supervised hypothesis-driven clinical research project?  Response options: Yes; No | | |
| (If 'Formal student practicum program' is marked) Mark all that you have completed while participating in a formal practicum program for clinical research. 1) clinical research- research involving human participants either through direct interaction or through the collection and analysis of blood, tissues, or other samples 2) Practicum- an experiential learning, hypothesis-driven, supervised investigation involving human subjects that applies and implements a clinical research design.  Response options: Submitted Institutional Review Board protocol; Institutional Review Board approval; Recruited/Recruiting participants; Data collection; Data analysis; Publication; Other | | |
| (If 'Research training through primary mentor' was marked) Mark all that you have completed with your primary research mentor in clinical research. Definition: clinical research- research involving human participants either through direct interaction or through the collection and analysis of blood, tissues, or other samples. Response options: Submitted Institutional Review Board protocol; Institutional Review Board approval; Recruited/Recruiting participants; Data collection; Data analysis; Publication; Other | | |
| **Clinical Research Domain** | **Cronbach Alpha** | **Clinical Research Appraisal Inventory Question** |
| Planning | 0.78 | 1. How confident are you in your ability to ask staff to leave the project team when necessary? |
|  |  | 2. How confident are you in your ability to set expectations and communicate them to project staff? |
| Designing and collecting | 0.79 | 3. How confident are you in your ability to design the best data analysis strategy for your study? |
|  |  | 4. How confident are you in your ability to determine an adequate number of subjects for your research project? |
| Funding | 0.85 | 5. How confident are you in your ability to describe the proposal review and award process for a major funding agency, such as the National Institutes of Health, National Science Foundation, or other foundation? ^a^ |
|  |  | 6. How confident are you in your ability to locate appropriate forms for a grant application? |
| Conceptualizing and collaborating | 0.68 | 7. How confident are you in your ability to identify faculty collaborators from within and outside the discipline who can offer guidance to the project? |
|  |  | 8. How confident are you in your ability to select a suitable topic area for study? |
| Protecting | 0.80 | 9. How confident are you in your ability to describe ethical concerns with the use of placebos in clinical research? |
|  |  | 10. How confident are you to apply the appropriate process for obtaining informed consent from research subjects? |
| Reporting, interpreting, and presenting | 0.91 | 11. How confident are you in your ability to write a discussion section for a research paper that articulates the importance of your findings relative to other studies in the field? |
|  |  | 12. How confident are you in your ability to write the results section of a research paper that clearly summarizes and describes the results, free of interpretative comments? |

^a^ Question was modified to remove acronyms.

Abbreviations: MS1, Medical School Year 1; MS2, Medical School Year 2; GS1, Graduate School Year 1; GS2, Graduate School Year 2; GS3, Graduate School Year 3; GS4, Graduate School Year 4; GS5, Graduate School Year 5; GS6, Graduate School Year 6; GS7, Graduate School Year 7; MS3, Medical School Year 3; MS4, Medical School Year 4.

**Appendix S2.** Proportion of clinical research training formats experienced by students in all years of training.

|  | **Year of Training** | | | | | | | | | | |
| --- | --- | --- | --- | --- | --- | --- | --- | --- | --- | --- | --- |
| **Training Format** | **MS1** | **MS2** | | **GS1** | **GS2** | **GS3** | **GS4** | **GS5-7** | | **MS3** | **MS4** |
| **None** | | | | | | | | | | | |
| No. in this study (%) | 34 (39.5) | 12 (24.0) | | 20 (20.4) | 15 (23.1) | 17 (23.3) | 23 (29.9) | 7 (35.0) | | 11 (19.0) | 11 (16.4) |
| 95% CI | 29.2-50.7 | 13.1-38.2 | | 12.9-29.7 | 13.5-35.2 | 14.2-34.6 | 20.0-41.4 | 15.4-59.2 | | 9.9-31.4 | 8.5-27.5 |
| **Didactics** | | | | | | | | | | | |
| No. in this study (%) | 32 (37.2) | 23 (46.0) | | 36 (36.7) | 19 (29.2) | 16 (21.9) | 29 (37.7) | 3 (15.0) | | 18 (31.0) | 18 (26.9) |
| 95% CI | 27.0-48.3 | 31.8-60.7 | | 27.2-47.1 | 18.6-41.8 | 13.1-33.1 | 26.9-49.4 | 3.2-37.9 | | 19.5-44.5 | 16.8-39.1 |
| **Research** | | | | | | | | | | | |
| No. in this study (%) | 6 (7.00) | 6 (12.0) | | 6 (6.1) | 10 (15.4) | 9 (12.3) | 10 (13.0) | 3 (15.0) | | 10 (17.2) | 10 (14.9) |
| 95% CI | 2.6-14.6 | 4.5-24.3 | | 2.3-12.9 | 7.6-26.5 | 5.8-22.1 | 6.4-22.6 | 3.2-37.9 | | 8.6-29.4 | 7.4-25.7 |
| **RP** | | | | | | | | | | | |
| No. in this study (%) | 0 (0.0) | 0 (0.0) | | 1 (1.0) | 0 (0.0) | 0 (0.0) | 1 (1.3) | 0 (0.0) | | 1 (1.7) | 1 (1.5) |
| 95% CI | 0-4.2 | 0-7.1 | | 0-5.6 | 0-5.5 | 0-4.9 | 0-7.0 | 0-16.8 | | 0.9.2 | 0-8.0 |
| **DR** | | | | | | | | | | | |
| No. in this study (%) | 9 (10.5) | 4 (8.0) | | 22 (22.4) | 13 (20.0) | 21 (28.8) | 10 (13.0) | 1 (5.0) | | 14 (24.1) | 12 (17.9) |
| 95% CI | 4.9-18.9 | 2.2-19.2 | | 14.6-32.0 | 11.1-31.8 | 18.8-40.6 | 6.4-22.6 | 0.1-24.9 | | 13.9-37.2 | 9.6-29.2 |
| **DP** | | |  | | |  | | |  | | |
| No. in this study (%) | 3 (3.5) | 2 (4.0) | | 4 (4.1) | 1 (1.5) | 3 (4.1) | 1 (1.3) | 1 (5.0) | | 2 (3.4) | 5 (7.5) |
| 95% CI | 0.7-9.9 | 0.5-13.7 | | 1.1-10.1 | 0-8.3 | 0.9-11.5 | 0-7.0 | 0.1-24.9 | | 0.4-11.9 | 2.5-16.6 |
| **DPR** | | | | | | | | | | | |
| No. in this study (%) | 2 (2.3) | 3 (6.0) | | 9 (9.2) | 7 (10.8) | 7 (9.6) | 3 (3.9) | 5 (25.0) | | 2 (3.4) | 10 (14.9) |
| 95% CI | 0.3-8.1 | 1.3-16.5 | | 4.3-16.7 | 4.4-20.9 | 3.9-18.8 | 0.8-11.0 | 8.7-49.1 | | 0.4-11.9 | 7.4-25.7 |
| **Total** | | | | | | | | | | | |
| No. in this study (%) | 86 (100) | 50 (100) | | 98 (100) | 65 (100) | 73 (100) | 77 (100) | 20 (100) | | 58 (100) | 67 (100) |

Abbreviations: RP, mentored clinical research plus clinical research practicum; DR, didactics plus mentored clinical research; DP, didactics plus clinical research practicum; DPR, didactics plus clinical research practicum plus mentored clinical research; SD, standard deviation; CI, confidence interval of the mean.
